# Morphological changes and induction of antifungal resistance in *Aspergillus fumigatus* due to different CO2 levels

**DOI:** 10.29252/cmm.3.3.21

**Published:** 2017-09

**Authors:** Sima Darabian, Sayed Jamal Hashemi, Sadegh Khodavaisy, Somayeh Sharifynia, Mohammad Kord, Maryam Akbari Dana, Farzad Aala, Sassan Rezaie

**Affiliations:** 1 Department of Medical Mycology and Parasitology, School of Public Health, International Campus, Tehran University of Medical Sciences, Tehran, Iran; 2 Department of Medical Mycology and Parasitology, School of Public Health, Tehran University of Medical Sciences, Tehran, Iran; 3 Clinical Tuberculosis and Epidemiology Research Center, National Research Institute of Tuberculosis and Lung Disease (NRITLD), Shahid Beheshti University of Medical Sciences, Tehran, Iran; 4 Department of Medical Mycology and Parasitology, Faculty of Medicine, Kurdistan University of Medical Sciences, Sanandaj, Iran

**Keywords:** *Aspergillus fumigatus*, Carbon dioxide, Itraconazole, Voriconazole

## Abstract

**Background and Purpose:**

Aspergillosis is one of the most common opportunistic fungal infections in immunocompromised and neutropenic patients. *Aspergillus fumigatus* (*A. fumigatus*) is the most common causative agent of this infection. Due to variable CO_2_ concentrations that pathogens are exposed to during the infection process and to understand the role of CO_2_, we examined the effects of various CO_2_ concentrations as one of the environmental factors on morphological changes and induction of antifungal resistance in *A. fumigatus*.

**Materials and Methods:**

*A. fumigatus* strains were cultured and incubated under 1%, 3%, 5%, and 12% CO_2_ atmospheres, each time for one, two, and four weeks. The control culture was maintained for one week without CO_2_ atmosphere. Morphological changes were investigated and antifungal susceptibility test was performed according to the recommendations of the Clinical and Laboratory Standards Institute (CLSI) M38-A2 document. The results of different CO_2_ atmospheres were compared with that of the control sample.

**Results:**

We found that 1%, 3%, 5%, and 12% CO_2_ atmospheres were associated with morphological colony changes. Macroscopically, the colonies were shallow dark green, smooth, crisp to powdery with reduced growth; microscopic examination revealed the absence of conidiation. The induction of antifungal resistance in the susceptible strains to itraconazole, voriconazole, and amphotericin B increased after exposure to 12% CO_2_ atmosphere and four weeks of incubation. The MIC values for itraconazole, voriconazole, and amphotericin B were 16 g/ml, 1 g/ml, and 16 g/ml, respectively. These values for the control group were 0.125 g/ml, 0.125 g/ml, and 2 g/ml, respectively.

**Conclusion:**

Exposure to different CO_2_ atmospheres induced morphological changes in *A. fumigatus*, it seems to increase the MIC values, as well. In parallel, resistance to both itraconazole and voriconazole was also observed.

## Introduction

*Aspergillus *species are widely distributed in the environment and have a wide range of manifestations from asymptomatic colonization or allergic reaction to invasive infection depending on host immunity [1, 2]. Invasive aspergillosis is the most severe manifestation with high mortality rate in immunocompromised patients [3, 4]. Amphotericin B (a member of the polyene antibiotics) and several azole-based medicines such as itraconazole, voriconazole, posaconazole, and isavuconazole are used for the treatment and prophylaxis of aspergillosis [5-7]. Recent reports from different European countries, the United States, South America, China, Japan, Iran, and India showed an increase in the frequency distribution of *A. fumigatus* isolates, indicating phenotypic resistance to itraconazole, voriconazole, and posaconazole [8-13].

Besides, specific point mutations in the *cyp51A* gene in combination with tandem repeats in the *cyp51A* promoter region are indicated as major environmentally derived mutations among azole-resistant *A. fumigatus* isolates. Additionally, *cyp51A*-independent resistance mechanisms, such as the upregulation of drug efflux transporters, have been recognized [14, 15]. 

Acquired resistance in *A. fumigatus* may be caused by long-term treatment and exposure of the fungal cells to azole fungicides used in agriculture. Along with these factors, environmental changes including the nutrient sources, humidity, temperature, and air pollution could potentially lead to physiological changes in microorganisms [16]. Atmospheric CO_2_ is considered an important agent for the biosphere homeostasis, which may cause changes in the physiological and morphological characteristics and the allergenic properties of saprophytic and pathogenic fungi. Such changes have been shown in *Cryptococcus neoformans* and *Candida albicans* during colonization and infection [17]. In addition, the effect of CO_2_ pressure on growth and aflatoxin production in *Aspergillus flavus* was previously reported [18-21]. Other studies revealed that increasing CO_2_ atmosphere in the culture medium could influence the pathogenicity of *A. fumigatus* [22, 23]. Due to the effectiveness of CO_2_ pressure as one of the environmental factors, we examined the effects of different CO_2_ concentrations on morphology and induction of antifungal resistance in *A. fumigatus*. 

## Materials and Methods

***A. fumigatus strain***

The wild-type strain of *A. fumigatus* (ATCC 1028) was cultured on Potato Dextrose Agar (PDA; Merck, Germany) and incubated at 35°C for 5-7 days. The suspension was prepared according to Clinical And Laboratory Standard Institute (CLSI M38-A2) protocol [24]. Briefly, fungal spores were harvested from 3 to 7 day-old cultures (in logarithmic growth phase) and mixed with distilled water containing 0.05% Tween 40; the mixture was adjusted at the final concentration of 1×10^6^ CFU/ml by spectrophotometer (530 nm) [24]. Afterwards, 200 μl of the obtained suspension was added to PDA culture medium plates [25]. The plates were incubated at 35°C for 1, 2, and 4 weeks under 1%, 3%, 5%, and 12% CO_2_ atmospheres separately. 

CO_2_ atmosphere was provided by using a 20-L cell culture CO_2_ incubator (SLS, USA). The desired CO_2_ injection was controlled by the calibrated automatic control panel in this incubator. The control culture was prepared under the same conditions without CO_2_ pressure.

***Macroscopic and microscopic examinations***

Macroscopic and microscopic criteria were investigated during culture under various CO_2 _atmospheres. The color and texture features of the colony (i.e., flat, granular, downy to powdery, and radial grooves) were studied during the incubation time. By using Lactophenol Cotton Blue, the microscopic criteria were considered to assess the morphological changes [26, 27]. All the cultures revealing morphological changes under CO_2_ atmospheres were inoculated in the Tryptic Soy Broth (TSB) medium (Merck, Germany) and stored at -20°C. After three weeks, the stocked cultures in TSB medium were sub-cultured in PDA medium and incubated at 35°C without CO_2_ atmosphere, and their morphological aspects were checked for reversibility evaluation. 

***Antifungal susceptibility test***

The minimum inhibitory concentrations (MICs) of three antifungal agents were evaluated according to CLSI (M38-A2) protocol [24, 28]. Itraconazole (Janssen Research Foundation, Beerse, Belgium), voriconazole (Pfizer, NY, USA), and amphotericin B (Bristol-Myers-Squibb, Woerden, the Netherlands) were obtained as reagent-grade powders dissolved in dimethyl sulfoxide (DMSO) and were diluted in standard RPMI 1640 medium (Sigma-Aldrich, St. Louis, MO, USA) buffered to pH 7.0 with 0.165 mol.L^-1^ morpholinepropanesulfonic acid buffer with L-glutamine without bicarbonate (MOPS, Sigma-Aldrich, St. Louis, MO, USA). The final concentrations of itraconazole, voriconazole, and amphotericin B ranged from 0.016 to 16 µg/ml. The plates were stored at -20°C prior to use [29]. All the cultured fungal cells under CO_2_ pressure were scraped after sporulation and the obtained spores were suspended in distilled water containing 0.05% Tween-40. The optical density (OD) of the supernatant was adjusted spectrophotometrically at 530 nm. The final concentrations of the stock inoculum suspensions were within the range of 0.5-4 × 10^4^ CFU/ml. Microdilution plates were incubated at 35°C and examined visually for MIC determination within 48 h of incubation. The MICs for itraconazole, voriconazole, and amphotericin B were defined as the concentration that fully inhibits fungal growth. The strain *Candida parapsilosis* ATCC 22019 was used as the negative quality control. All the tests were performed in duplicate.

This study was approved by the Ethics Committee of Tehran University of Medical Sciences, Tehran, Iran (code: IR.TUMS.SPH.REC.1396.2289).

## Results

**Figure 1:**
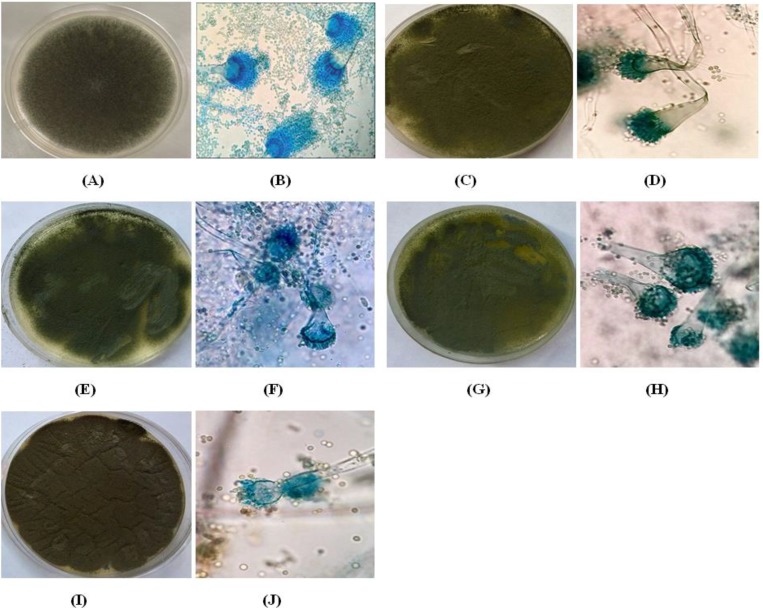
Photographs of macroscopic morphological changes of *A. fumigatus* following exposure to different levels of CO_2_ after four weeks.

We observed significant changes in the macroscopic and microscopic characteristics of *A. fumigatus* wild-type in different situations (CO_2_ atmosphere and incubation duration). The initial macro- and micro-colony changes occurred in the presence of low CO_2_ pressure (1%). However, the minimum of such changes were observed under 3% and 5% CO_2_ atmospheres after one, two, and four weeks, while maximum changes happened under 12% CO_2_ atmosphere after four weeks. The major macroscopic changes appeared in the culture media that included shallow colonies with dark green color, smooth and crisp to powdery appearance, and reduced growth. However, the major microscopic changes appeared in exposed fungal cells with short conidiophores, deformed vesicles, and declined conidia (Figure 1). These results were obtained by comparison with the control colonies, which were blue-green, fast-growing, fluffy to powdery colonies with normal conidiophores, arranged vesicles, phialides, metulae, and smooth hyaline conidia (Figure 1). The reversibility of morphological changes was reviewed through culture of the samples after four weeks in the absence of CO_2_ atmosphere. The obtained results indicated that *A. fumigatus* could maintain the described changes even in the absence of CO_2_ atmosphere. 

The MIC values for itraconazole, voriconazole, and amphotericin B under various CO_2 _atmospheres and incubation times are listed in Table 1. The MIC values after 48 h of incubation increased with elevated CO_2_ pressure and prolonged incubation time. Under 12% CO_2_ atmosphere and after four weeks of incubation, the MIC values for itraconazole, voriconazole, and amphotericin B were 16 g/ml, 1 g/ml, and 16 g/ml, respectively. However, these values were 0.125 g/ml, 0.125 g/ml, and 1 g/ml, respectively, in the control group (Table 1).

(A) Macroscopic examination with the original colonie; (B) microscopic examination with the normal features of conidiation; (C, D) inception of changes were observed under 1% CO_2_ atmosphere, (E, F) 3% CO_2_ atmosphere, (G, H) 5% CO_2_ atmosphere, and (I, J) the most changes occurred under 12% CO_2 _atmosphere.

**Table 1:** In vitro susceptibility of *A. fumigatus* to itraconazole, voriconazole, and amphotericin B under different CO_2_ atmospheres

**Strain**	**CO**_2_ ** level**	**Time (week)**	**Minimum inhibitory concentration (g/ml)**		
			**ITR**	**VOR**	**AMB**
*A. fumigatus* (ATCC 1028)	1% CO_2_	1	0.125	0.5	4
		2	0.5	0.125	4
		4	1	0.125	8
	3% CO_2_	1	1	0.5	8
		2	1	0.125	8
		4	1	0.5	8
	5% CO_2_	1	0.5	0.5	8
		2	2	0.5	8
		4	8	0.5	16
	12% CO_2_	1	1	0.5	8
		2	2	0.5	8
		4	16	1	16
	Control cultures		0.125	0.125	1

Abbreviations: ITR: itraconazole; VOR: voriconazole; AMB: amphotericin B

## Discussion

*A. fumigatus* is one of the most important fungal pathogens and the main causative agent of invasive aspergillosis. This species is affected by environmental conditions such as CO_2_ [23, 30]. Studies showed that CO_2_ could significantly affect fungal physiology and morphology [31]. Previous studies on the effects of CO_2_ atmosphere on pathogenesis and metabolism of *C. albicans* and *Cryptococcus neoformans* revealed the induction of invasion to blood in *C. albicans* cells under 5% CO_2_ atmosphere. Besides, filamentation and pseudohyphal formation were reported to be induced in such cells [25, 32, 33]. Capsular polysaccharide synthesis of *C. neoformans* was stimulated after exposure to 5% CO_2_ atmosphere [23, 34-36]. Another study indicated the occurrence of arthroconidium formation, a resistance factor in *T. rubrum*, after 10 days of incubation on Sabouraud Dextrose Agar medium at 37°C under 10% CO_2_ atmosphere [37]. Some investigations have considered the effect of CO_2_ on fungal growth and found a general inhibitory effect under CO_2_ atmospheres [31]. Coelho et al. reported that high CO_2_ pressure could have detrimental effects on growth and metabolism of yeasts, and therefore, could contribute to the inactivation of *Saccharomyces cerevisiae* cells [38, 39]. Due to its antimicrobial activity, CO_2 _can be particularly used for storing food commodities through the inactivation of the microorganisms [40]. Changed CO_2_ atmosphere, carbonic anhydrases (CAs), and C: N ratio have been reported to affect growth by influencing the production of proteins and the content of fungal spores [41, 42]. The effects of CO_2_ on morphology, growth, and citric acid production in *A. niger* were also reported by McIntyre and McNeil [31].

 Recent investigations indicated that *A. fumigatus* and *A. flavus* spores grown under varying CO_2_ atmosphere showed higher allergenicity [22, 32]. In line with the previous investigations, we appraised the effect of CO_2_ on morphological changes in *A. fumigatus*. The obtained results indicated that CO_2_ atmosphere could enhance some morphological (macroscopic and microscopic) changes such as sporulation, vesicle deformation, and the development of *Chlamydia* spores in *A. fumigatus* strains. The majority of the mentioned changes were also indicated to occur under 12% CO_2_ atmosphere and after four weeks of incubation. Previous reports revealed the capability of pathogenic *Aspergillus* species in growth under CO_2_ atmosphere; they also showed that this capacity might affect the pathogenicity and antifungal activity of these species [20]. Our findings may help with deeper understanding of this process. Besides, variation in CO_2_ atmosphere in environments where *A.* *fumigatus* is able to grow reveals the importance of effective factor and CO_2_ absorb regulator genes in these fungi. 

Our results manifested that MICs of itraconazole, voriconazole, and amphotericin B against *A. fumigatus* increased near high CO_2_ environments. However, the mechanism of resistance induction by CO_2_ remains inconspicuous. Some medicines and environmental factors have been used for the induction of drug resistance in *A. fumigatus* [43]. In some studies, different types of resistance mechanisms, including changes in the target lanosterol-14-demethylase and induction of high-capacity drug efflux pumps, have been shown to promote drug resistance following UV-induced mutations [44-46]. In the study of Escribano et al., the MICs of itraconazole, voriconazole, and posaconazole against *A. fumigatus* cells were determined before and after exposure of the fungal cells to itraconazole. The obtained results indicated that the final MICs were substantially higher than the initial ones [47]. Faria-Ramos et al. investigated cross-resistance in *A. fumigatus* to clinical azoles after the exposure of fungal cells to prochloraz (an agronomical azole) and found that resistance to prochloraz increased after the initial exposure [48]. In addition, prochloraz exposure caused some morphological changes in fungal colony such as changing the appearance to white-colored colonies, losing the typical pigmentation, and absence of conidiation [48]. Our results also indicated the macroscopic and microscopic morphological changes in *A. fumigatus* due to CO_2_ pressure. However, these morphological changes were not similar to the alterations described by Faria-Ramos et al. 

The results of these studies indicate that *A. fumigatus* can be resistant to antifungal compounds due to antifungal agents in medicine, fungicides used in agriculture, and environmental factors. The results of this study also indicated that high concentrations of CO_2 _could be considered as an environmental factor affecting the occurrence of drug resistance in fungi.

## Conclusion

Different levels of CO_2_ exposure induced morphological changes in *A. fumigatus*, an evident increase in MIC values, and the development of cross-resistance to itraconazole and voriconazole. Our next step is assessing the underlying molecular resistance mechanisms in these induced resistant strains, as well as in isolates with naturally high MIC values in different levels of CO_2_ and evaluating resistance to antifungal drugs without any prior in vitro induction.

## References

[1] Rocchi S, Reboux G, Millon L (2015). Azole resistance with environmental origin: what alternatives for the future?. J Mycol Med.

[2] Abdolrasouli A, Rhodes J, Beale MA, Hagen F, Rogers TR, Chowdhary A (2015). Genomic context of azole resistance mutations in Aspergillus fumigatus determined using whole-genome sequencing. MBio.

[3] Denning DW, Pleuvry A, Cole DC (2013). Global burden of allergic bronchopulmonary aspergillosis with asthma and its complication chronic pulmonary aspergillosis in adults. Med Mycol.

[4] Meersseman W, Lagrou K, Maertens J, Van Wijngaerden E (2007). Invasive aspergillosis in the intensive care unit. Clin Infect Dis.

[5] Maertens JA, Blennow O, Duarte RF, Muñoz P (2016). The current management landscape: aspergillosis. J Antimicrob Chemother.

[6] Patterson TF, Thompson GR 3rd, Denning DW, Fishman JA, Hadley S, Herbrecht R (2016). Practice guidelines for the diagnosis and management of Aspergillosis: 2016 update by the infectious diseases society of America. Clin Infect Dis.

[7] Denning DW, Venkateswarlu K, Oakley KL, Anderson MJ, Manning NJ, Stevens DA (1997). Itraconazole resistance in Aspergillus fumigatus. Antimicrob Agents Chemother.

[8] Seyedmousavi S, Hashemi SJ, Zibafar E, Zoll J, Hedayati MT, Mouton JW (2013). Azole-resistant Aspergillus fumigatus, Iran. Emerg Infect Dis.

[9] Nabili M, Shokohi T, Moazeni M, Khodavaisy S, Aliyali M, Badiee P (2016). High prevalence of clinical and environmental triazole-resistant Aspergillus fumigatus in Iran: is it a challenging issue?. J Med Microbiol.

[10] Snelders E, Huis In 't Veld RA, Rijs AJ, Kema GH, Melchers WJ, Verweij PE (2009). Possible environmental origin of resistance of Aspergillus fumigatus to medical triazoles. Appl Environ Microbiol.

[11] Gonçalves SS, Souza AC, Chowdhary A, Meis JF, Colombo AL (2016). Epidemiology and molecular mechanisms of antifungal resistance in Candida and Aspergillus. Mycoses.

[12] Escribano P, Peláez T, Muñoz P, Bouza E, Guinea J (2013). Is azole resistance in Aspergillus fumigatus a problem in Spain?. Antimicrob Agents Chemother.

[13] Chowdhary A, Kathuria S, Xu J, Meis JF (2013). Emergence of azole-resistant Aspergillus fumigatus strains due to agricultural azole use creates an increasing threat to human health. PLoS Pathog.

[14] Shalhoub S, Luong ML, Howard SJ, Richardson S, Singer LG, Chaparro C (2015). Rate of cyp51A mutation in Aspergillus fumigatus among lung transplant recipients with targeted prophylaxis. J Antimicrob Chemother.

[15] Wang HC, Huang JC, Lin YH, Chen YH, Hsieh MI, Choi PC (2018). Prevalence, mechanisms, and genetic relatedness of the human pathogenic fungus Aspergillus fumigatus exhibiting resistance to medical azoles in the environment of Taiwan. Environ Microbiol.

[16] da Silva Ferreira ME, Capellaro JL, dos Reis Marques E, Malavazi I, Perlin D, Park S (2004). In vitro evolution of itraconazole resistance in Aspergillus fumigatus involves multiple mechanisms of resistance. Antimicrob Agents Chemother.

[17] Sheth CC, Johnson E, Baker ME, Haynes K, Mühlschlegel FA (2005). Phenotypic identification of Candida albicans by growth on chocolate agar. Med Mycol.

[18] Hedayati MT, Pasqualotto AC, Warn PA, Bowyer P, Denning DW (2007). Aspergillus flavus: human pathogen, allergen and mycotoxin producer. Microbiology.

[19] Ellis W, Smith J, Simpson BK, Khanizadeh S, Oldham JH (1993). Control of growth and aflatoxin production of Aspergillus flavus under modified atmosphere packaging (MAP) conditions. Food Microbiol.

[20] Hall LA, Denning DW (1994). Oxygen requirements of Aspergillus species. J Med Microbiol.

[21] Gilbert MK, Mack BM, Payne GA, Bhatnagar D (2016). Use of functional genomics to assess the climate change impact on Aspergillus flavus and aflatoxin production. World Mycotoxin J.

[22] Lang-Yona N, Levin Y, Dannemiller KC, Yarden O, Peccia J, Rudich Y (2013). Changes in atmospheric CO2 influence the allergenicity of Aspergillus fumigatus. Glob Change Biol.

[23] Tobal JM, Balieiro ME (2014). Role of carbonic anhydrases in pathogenic micro-organisms: a focus on Aspergillus fumigatus. J Med Microbiol.

[24] Wayne P (2008). Reference method for broth dilution antifungal susceptibility testing of filamentous fungi Approved standard-second edition. Clin Lab Stand Instit.

[25] Sasani E, Khodavaisy S, Agha Kuchak Afshari S, Darabian S, Aala F, Rezaie S (2016). Pseudohyphae formation in Candida glabrata due to CO2 exposure. Curr Med Mycol.

[26] Latgé JP (1999). Aspergillus fumigatus and aspergillosis. Clin Microbiol Rev.

[27] McClenny N (2005). Laboratory detection and identification of Aspergillus species by microscopic observation and culture: the traditional approach. Med Mycol.

[28] Wayne PA (2011). Clinical and Laboratory Standards Institute; 2011 CLSI Performance standards for antimicrobial susceptibility testing 20th Informational Supplement CLSI document M100-S21. Clin Lab Stand Instit.

[29] Khodavaisy S, Badali H, Hashemi SJ, Aala F, Nazeri M, Nouripour-Sisakht S (2016). In vitro activities of five antifungal agents against 199 clinical and environmental isolates of Aspergillusflavus, an opportunistic fungal pathogen. J Mycol Med.

[30] Mousavi B, Hedayati MT, Hedayati N, Ilkit M, Syedmousavi S (2016). Aspergillus species in indoor environments and their possible occupational and public health hazards. Curr Med Mycol.

[31] Papagianni M (2004). Fungal morphology and metabolite production in submerged mycelial processes. Biotechnol Adv.

[32] Bahn YS, Cox GM, Perfect JR, Heitman J (2005). Carbonic anhydrase and CO2 sensing during Cryptococcus neoformans growth, differentiation, and virulence. Curr Biol.

[33] Du H, Guan G, Xie J, Cottier F, Sun Y, Jia W (2012). The transcription factor Flo8 mediates CO2 sensing in the human fungal pathogen Candida albicans. Mol Biol Cell.

[34] Mogensen EG, Janbon G, Chaloupka J, Steegborn C, Fu MS, Moyrand F (2006). Cryptococcus neoformans senses CO2 through the carbonic anhydrase Can2 and the adenylyl cyclase Cac1. Eukaryot Cell.

[35] Klutts JS, Doering TL (2008). Cryptococcal xylosyltransferase 1 (Cxt1p) from Cryptococcus neoformans plays a direct role in the synthesis of capsule polysaccharides. J Biol Chem.

[36] Kim MS, Ko YJ, Maeng S, Floyd A, Heitman J, Bahn YS (2010). Comparative transcriptome analysis of the CO2 sensing pathway via differential expression of carbonic anhydrase in Cryptococcus neoformans. Genetics.

[37] Yazdanparast SA, Barton RC (2006). Arthroconidia production in Trichophyton rubrum and a new ex vivo modelof onychomycosis. J Med Microbiol.

[38] Coelho M, Belo I, Pinheiro R, Amaral A, Mota M, Coutinho J (2004). Effect of hyperbaric stress on yeast morphology: study by automated image analysis. Appl Microbiol Biotechnol.

[39] Shimoda M, Cocunubo‐Castellanos J, Kago H, Miyake M, Osajima Y, Hayakawa I (2001). The influence of dissolved CO2 concentration on the death kinetics of Saccharomyces cerevisiae. J Appl Microbiol.

[40] Shimoda M, Yamamoto Y, Cocunubo‐Castellanos J, Tonoike H, Kawano T, Ishikawa H (1998). Antimicrobial effects of pressured carbon dioxide in a continuous flow system. J Food Sci.

[41] Rhodes JC (2006). Aspergillus fumigatus: growth and virulence. Med Mycol.

[42] Mavridou E, Brüggemann RJ, Melchers WJ, Mouton JW, Verweij PE (2010). Efficacy of posaconazole against three clinical Aspergillus fumigatus isolates with mutations in the cyp51A gene. Antimicrob Agents Chemother.

[43] Krishnan S, Manavathu EK, Chandrasekar PH (2009). Aspergillus flavus: an emerging non fumigatus Aspergillus species of significance. Mycoses.

[44] Sanglard D, Ischer F, Koymans L, Bille J (1998). Amino acid substitutions in the cytochrome P-450 lanosterol 14α-demethylase (CYP51A1) from azole-resistant Candida albicans clinical isolates contribute to resistance to azole antifungal agents. Antimicrob Agents Chemother.

[45] Osherov N, Kontoyiannis DP, Romans A, May GS (2001). Resistance to itraconazole in Aspergillus nidulans and Aspergillus fumigatus is conferred by extra copies of the A nidulans P-450 14α-demethylase gene, pdmA. J Antimicrob Chemother.

[46] Sanglard D, Kuchler K, Ischer F, Pagani JL, Monod M, Bille J (1995). Mechanisms of resistance to azole antifungal agents in Candida albicans isolates from AIDS patients involve specific multidrug transporters. Antimicrob Agents Chemother.

[47] Escribano P, Recio S, Peláez T, González-Rivera M, Bouza E, Guinea J (2012). In vitro acquisition of secondary azole resistance in Aspergillus fumigatus isolates after prolonged exposure to itraconazole: presence of heteroresistant populations. Antimicrob Agents Chemother.

[48] Faria-Ramos I, Farinha S, Neves-Maia J, Tavares PR, Miranda IM, Estevinho LM (2014). Development of cross-resistance by Aspergillus fumigatus to clinical azoles following exposure to prochloraz, an agricultural azole. BMC Microbiol.

